# Evaluation of Physical Properties of Generic and Branded Travoprost Formulations

**DOI:** 10.5005/jp-journals-10008-1201

**Published:** 2016-08-05

**Authors:** Meenakshi Wadhwani, Sanjay K Mishra, Dewang Angmo, Thirumurthy Velpandian, Ramanjit Sihota, Ankita Kotnala, Shibal Bhartiya, Tanuj Dada

**Affiliations:** 1 Senior Research Officer, Glaucoma Research Facility and Clinical Services Dr. Rajendra Prasad Centre for Ophthalmic Sciences, All India Institute of Medical Sciences, New Delhi, India; 2 Associate Professor, Department of Opthalmology, Army Research and Referral Hospital, New Delhi, India; 3 Senior Research Associate, Glaucoma Research Facility and Clinical Services Dr. Rajendra Prasad Centre for Ophthalmic Sciences, All India Institute of Medical Sciences, New Delhi, India; 4 Professor, Department of Ocular Pharmacology, Dr. Rajendra Prasad Centre for Ophthalmic Sciences, All India Institute of Medical Sciences New Delhi, India; 5 Professor, Glaucoma Research Facility and Clinical Services Dr. Rajendra Prasad Centre for Ophthalmic Sciences, All India Institute of Medical Sciences, New Delhi, India; 6 PhD Student, Department of Ocular Pharmacology, Dr. Rajendra Prasad Centre for Ophthalmic Sciences, All India Institute of Medical Sciences New Delhi, India; 7 Senior Consultant, Department of Opthalmology, Fortis Memorial Research Institute, Gurgaon, Haryana, India; 8 Professor, Glaucoma Research Facility and Clinical Services Dr. Rajendra Prasad Centre for Ophthalmic Sciences, All India Institute of Medical Sciences, New Delhi, India

**Keywords:** Branded drugs, Ophthalmic generics, Prostaglandin analogs, Travoprost.

## Abstract

**Purpose:** Comparative evaluation of pharmaceutical characteristics of three marketed generic *vs* branded travoprost formulations.

**Materials and methods:** Three generic travoprost formulations and one branded (Travatan without benzalkonium chloride) formulation (10 vials each), obtained from authorized agents from the respective companies and having the same batch number, were used. These formulations were coded and labels were removed. At a standardized room temperature of 25°C, the drop size, pH, relative viscosity, and total drops per vial were determined for Travatan (Alcon, Fort Worth, TX, USA) and all the generic formulations. Travoprost concentration in all four brands was estimated by using liquid chromatography-coupled tandem mass spectrometry LCMS.

**Results:** Out of the four formulations, two drugs (TP 1 and TP 4) were found to follow the United States Pharmacopoeia (USP) limits for ophthalmic formulation regarding drug concentration, while the remaining two drugs failed due to the limits being either above 110% (TP 2) or below 90% (TP 3). Two of them (TP 1 and TP 2) had osmolality of 313 and 262 mOsm respectively, which did not comply with the osmolality limits within 300 mOsm (+ 10%). The pH of all the formulations ranged between 4.7 and 5.9, and the mean drop size was 30.23 ± 6.03 uL. The total amount of drug volume in the bottles varied from 2.58 ± 0.15 to 3.38 ± 0.06 mL/bottle.

**Conclusion:** There are wide variations in the physical properties of generic formulations available in India. Although some generic drugs are compliant with the pharmacopeia standards, this study underscores the need for a better quality control in the production of generic travoprost formulations.

**How to cite this article:** Wadhwani M, Mishra SK, Angmo D, Velpandian T, Sihota R, Kotnala A, Bhartiya S, Dada T. Evaluation of Physical Properties of Generic and Branded Travoprost Formulations. J Curr Glaucoma Pract. 2016;10(2):49-55.

## INTRODUCTION

Glaucoma it is the most common cause of irreversible blindness globally, and it is estimated that more than 3 million people are blind due to glaucoma.^1^ The only risk factor for glaucoma that has been proven to be amenable to intervention is intraocular pressure (IOP), the reduction of which is known to prevent glaucoma progression. Topical prostaglandin analogs have become the first line ocular hypotensive therapy for the treatment of glaucoma due to their efficacy, safety, and patient acceptability due to once-a-day dosage.^2^ Given that glaucoma is a chronic disease which cannot be cured but requires lifelong therapy, the physical properties of these ocular hypoten-sive agents are of great concern to the ophthalmologists as well as the patients.

Like its previous congeners, bimatoprost and latano-prost, travoprost is also used as first line therapy in the management of glaucoma. Travoprost 0.004% is a prodrug of a prostaglandin F2 alpha (PGF2α) and effectively reduces IOP in glaucoma and normal subjects. It is generally recognized that PGF2α analogs induce the synthesis of matrix metalloproteases (MMPs) in the ciliary body and sclera and increase uveoscleral outflow (“pressure insensitive”).^3,4^

Given the increasing economic burden of disease in an ageing demographic, health care authorities are increasingly supporting the use of generic substitution. Many developing countries do not have the resources or expertise to carry out appropriate quality control tests resulting in widespread distribution of substandard, or even counterfeit, drugs. Even in countries where procedures are well-regulated, substandard drugs reach the market from time to time.^5^

Counterfeiting can apply to both generic and branded drugs. The World Health Organization has defined counterfeit drugs as “[a] medicine which is deliberately and fraudulently mislabeled with respect to identity and or/ source.” It may include products with correct ingredients or with wrong ingredients, without active ingredients, with insufficient active ingredients, or with false packaging.^6^

Despite the regulatory requirements on generics for bioequivalence and presumed therapeutic equivalence, there are compositional differences between generic and brand-name drugs which can affect both efficacy and safety profiles. To the best of our knowledge, a comparative evaluation of physical properties of generics *vs* branded travoprost formulations has not been performed previously, and we initiated this pilot study to compare the physical properties, absolute drug concentration, and unit dosage of three generics of travoprost and branded Travatan with the acceptable standards.

## MATERIALS AND METHODS

Three branded generics of travoprost and one branded (Travatan) formulation (10 vials each) were obtained from authorized chemists. Each of the 10 vials of 4 travoprost formulations had the same batch number. These formulations were coded and labels were removed to eliminate bias. They were subjected to analysis at a standardized room temperature of 25°C in the ocular pharmacology department of a university hospital. The branded travo-prost used was benzalkonium chloride (BAK)-free. Out of the three generic formulations, two were BAK-free while one contained BAK (Table 1).

Travoprost pure compound and internal standard (sul-fadimethoxine) were purchased from Caymen Chemicals, Caymen Chemicals company (1180 E. Ellsworth Ann Arbor, MI United States). Mass grade formic acid and acetonitrile were purchased from Merck, Germany. All other chemicals and solvents used were of the highest analytical grades available.

A stock solution of travoprost was prepared in pure methanol to arrive at a concentration of 1 mg/mL. This stock solution was appropriately diluted with 50% methanol, containing 0.1% formic acid, to reach the working standards of required concentration on the day of the experiment. A calibration curve was plotted with concentrations ranging from 3.9 to 500 ng/mL. Stock solution containing 100 ng/mL sulfadimethoxine in pure methanol was used as the internal standard (IS).

### Liquid Chromatography-mass Spectrometry (LCMS)

Chromatography separation was achieved using ultra-high performance liquid chromatography (UPLC) (Thermo Surveyor system, Thermo Electron Corp, Waltham, MA, USA) with a quaternary pump connected to an online degasser and photodiode array detector (PDA). Ultrahigh performance liquid chromatography was coupled with triple quadruple tandem mass spec-troscopy (4000 Q-Trap, ABS Biosystems, Foster City CA, USA). ChromQuest software version 5.1 was used to control all parameters of UPLC. For analytical separation of latanoprost, a LiChroCART 55-4 Purosphere STAR RP 18e 3 μm column was used.

**Supplementary Graph 1:**
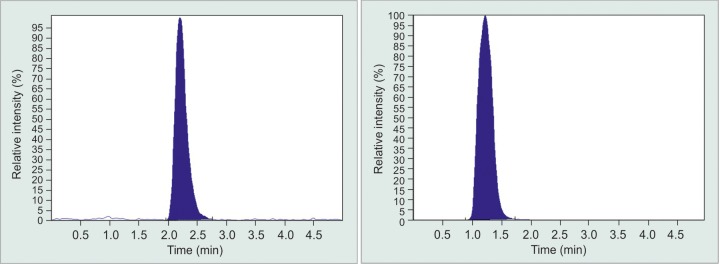
Travoprost peaks in LCMS chromatogram. It contains online-only supplementary material

The isocratic mobile phase consisted of acetonitrile containing 0.1% formic acid (A) and Water containing 0.1% formic acid (B) in the ratio of 7:3; and was pumped at the rate of 0.5 mL/minute. The autosampler tray and the column were kept at ambient temperature. Twenty microliter of sample was injected into the UPLC with a run time of 5 minutes. Tandem Mass spectrometric detection of analyte and internal standard (IS) was carried out on an Applied Bio Systems 4000 triple quadruple instrument (ABS Biosystems, Foster City CA, USA) equipped with a TurboIonSpray (ESI) source that operated in the positive ion mode. Quantification was performed using multiple reaction monitoring (MRM) mode, based on molecular adduct and its fragment ion latanoprost having the transition of m/z 433.3/319.2 and travoprost having transitions of 501.0/460.3 (Supplementary Graph 1). The transition for sulfadimethoxine was m/z 311/156. Data acquisition and integration was performed by Analyst 1.4.2. software (ABS Biosystems, Foster City CA, USA).

### Sample Preparation

A 20 μL of standards or samples were mixed with 200 μL of extraction solvent, i.e., pure methanol containing 100 ng/mL sulfadimethoxine. The mixture was vortexed for 1 minute and centrifuged for 10 minutes at 7840 gm. The resultant supernatant was subjected to analysis.

### Physical Property

Osmolality of the formulations was determined using μOSMETTE (Micro-osmometer) while pH of the formulation was determined using EUTECH instrument pH 510 pH/mVPC meter (Thermoscientific, Waltham, MA, USA). Relative viscosity was determined using Ostwald’s viscometer (Rheolab QC, Antonpaar, GmbH, Strassc 208054, Austria) while specific gravity and drop size were calculated using calibrated microbalance (Sartorius, CPA225D, AG, Gottingen, Germany). All experiments were performed at standard ambient temperature and humidity conditions.

Statistical analysis was performed using Statistical Package for the Social Sciences (SPSS) (IBM^®^SPSS^®^ Statistics version 20). Data are expressed in mean ± SD. Continuous variables were compared among the group by one way analysis of variance (ANOVA) followed by posthoc analysis using Dunnett test [taking Travatan as standard, as it conformed to the United States Pharmacopeia (USP) limits]. A p-value of < 0.05 was considered statistically significant.

**Table 1:** **Table 1**: Comparison of various physical parameters of three generic and one branded travoprost formulations

		*Drug*		*Drug concentration (mean ± SD)*															
*Drug formulations*		*(Preservative)*		*% purity*		*(μg/mL)*		*(μg/drop)*		*Amount of solution per* * bottle (mL) (mean ± SD)*		*Drop Size (μL)* *(mean ± SD)*		*Osmolality* * (mOsm)* * (mean ± SD)*		*pH* * (mean ± SD)*		*Specific gravity* * (mean ± SD)*	
TP1 (Travo)		Ionic buffer system with boron polyoxol complex		0.0037± 0.0031 (91.79±7.89)		36.21 ±0.86		1.42±0.02		3.38±0.06		38.9±3.2		313.0±1.0		5.9±0.0		1.00±0.003	
TP2 (Tovaxo)		Benzalkonium chloride (0.015%)		0.0056±0.0002 (140.4±6.3)		56.62±0.40		1.44±0.01		2.58±0.15		25.8±3.7		262.0±1.0		4.7±0.25		0.99±0.005	
TP3 (Xovatra)		Isotonic buffered aqueous vehicle preserved with ionic buffer system		0.0034±0.0003 (86.71 ±8.072)		34.51 ±0.61		1.02±0.01		3.13±0.28		29.7±3.3		305.3±3.0		5.7±0.26		(0.99±0.000)	
TP4 (Travatan)		Polyquaternium-1		0.0038±0.0005 (95.37± 13.34)		38.14±1.05		1.01 ±0.00		3.00±0.33		26.5±4.3		308.0±1.7		5.8±0.06		0.99±0.003	
Overall between				0.000*		0.000*		0.000*		0.000*		0.000*		0.000*		0.000*		0.000*	
group p-value																			
Posthoc analysis		TP1 *vs* TP4		0.723		0.000*		0.000*		0.000*		0.000*		0.000*		0.745		0.000*	
p-value		TP2 *vs* TP4		0.000*		0.000*		0.000*		0.000*		0.591		0.000*		0.000*		0.000*	
		TP3 *vs* TP4		0.111		0.000*		0.068		0.274		0.006*		0.001*		0.645		0.001	

*p<0.05, statistically significant

**Graph 2:**
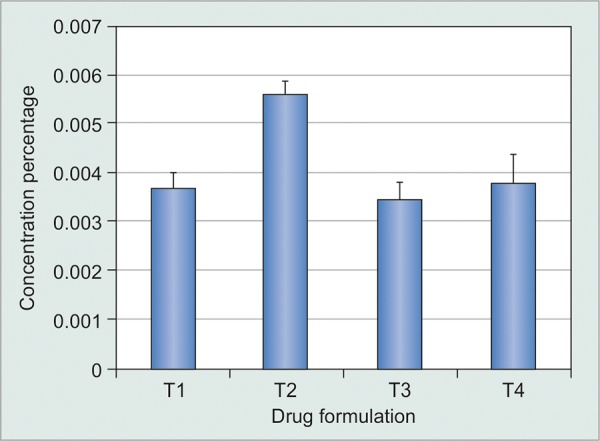
Percentage concentration of various travoprost formulations

**Graph 3:**
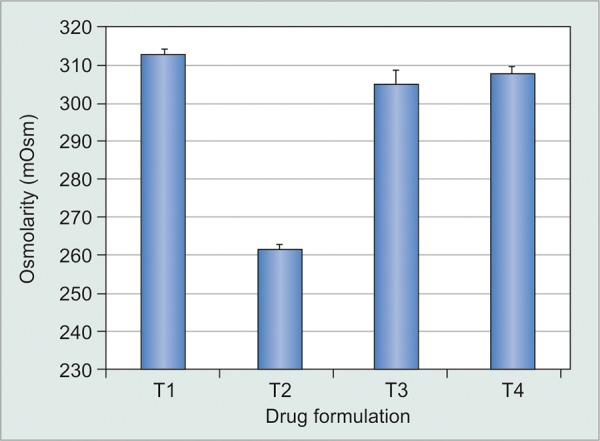
Osmolarity of various travoprost formulations

## RESULTS

Samples were analyzed at the indicated dilutions. The various formulations were labeled as TP1-TP4. The concentration of drugs varied from 34.51 ± 0.61 to 56.62 ± 0.40 μg/mL (0.0034 ± 0.0003-0.0056 ± 0.0002%) as compared to the standard labeled concentration of 40 μg/mL (0.004%) on the travoprost vials (Graph 2). Two of them (TP1 and TP4) were found to follow the USP limits for ophthalmic formulation regarding drug concentration, while the remaining 2 drugs failed due to the limits being either above 110% (TP2) or below 90% (TP3) of USP limits.

The mean osmolality of two formulations (TP1 and 2) were 313.0 ± 1.0 and 262.0 ± 1.0 mOsm, which did not comply with the USP limits of 300 mOsm (+ 10%). The mean osmolality of TP3 was 305.3 ± 3.0 mOsm and TP4 (Travatan) was 308.0 ± 1.7 mOsm, which complied with the standard osmolality limits (Graph 3). The pH of the formulations were TP1 = 5.9 ± 0.0, TP2 = 4.7 ± 0.25, TP3 = 5.7 ± 0.26, and TP4=5.8 ± 0.06. The mean drop size in TP1-TP4 was 38.9 ± 3.2 μL, 25.8 ± 3.7 μL, 29.7 ± 3.3 μL, and 26.5 ± 4.3 μL respectively. The mean content of bottles were 3.38 ± 0.06 mL/bottle in TP1, 2.58 ± 0.15 mL/bottle in TP2, 3.13 ± 0.28 mL/bottle in TP3, and 3.00 ± 0.33 mL/ bottle in TP4 (Table 1).

## DISCUSSION

It is essential to remember that bioequivalence is the main criteria for any generic drug to be approved for use *in vivo.* The Food and Drug Administration (FDA) defines therapeutic equivalence as having the same clinical effect and safety profile as the reference listed drugs which mandates that the amount of absorption of generic drug must be within a certain range of their counterpart branded drug.^7^ This is of great concern to the treating ophthalmologists as generic drugs are released into the market without randomized controlled trials on efficacy and safety, and the processes used for production and standardization may not be uniform across companies.^5-7^

Travoprost is a pro-drug of prostaglandin F_2_a analog approved for glaucoma. Travatan (Alcon, Fort Worth, TX, USA) is a sterile ophthalmic formulation supplied as an isotonic, buffered aqueous solution with a pH of 6.4 to 7.0 and an osmolality of 265 to 320 mOsmol/kg, with polyquaternium-1 (POLYQUAD) as preservative. As per the packaging insert, 1 mL is capable of delivering 40 μg of travoprost. It is supplied in the volume of 2.5 mL solution in a 4 mL natural syndiotactic polypropylene (sPP) oval bottle with a polypropylene (PP) natural dispensing plug and a white PP closure. The completed package is placed into a foil overwrap, which also provides a tamper evident feature.

Even though Travatan is known for its safety and efficacy in glaucoma management, its ocular side effects include conjunctival hyperemia, increased iris pigmentation, and lengthening of eyelashes, as well as increase in incidence of cystoid macular edema and uveitis in predisposed patients.^8,9^

Systemic generic medications are required to show bioequivalence (similar absorption characteristics) before gaining acceptance in the market. The bioequivalence of ophthalmic generic medications cannot be assessed in terms of absorption into the eye.

Although there are strict government regulations for generics by way of drug concentrations, the bio-equivalence may be variable due to conditions of pH and osmolarity in terms of both efficacy and side effects, since minor differences in formulation may affect absorption as well as the comfort and consistency of the eye drop.

Our study observed that the concentration of travo-prost varied between different commercially available formulations. It therefore stands to reason that since the concentration of the drug is more than the labeled concentration, then the side effect profile of the drug is likely to be more. On the contrary, if the concentration is less, then the potency of the drug may be in question. To ensure that a generic ophthalmic product has the “same” concentration, approval of generic ophthalmic products is based on having the generic ophthalmic match each active and inactive ingredient to within ± 5% of the innovator target formulation.^10^ The concentration and other pharmacokinetic properties of not only Travatan but also one of the generic Indian formulation followed the USP norms (TP1). The mean concentration of branded travoprost was 38.14 ± 1.05 ug/mL. Out of the three generics studied, the concentration of TP1 was 36.21 ± 0.86 ug/mL whereas the other two had either more than 40 (TP2) or less than 35 ug/mL (TP3) as compared to the claimed concentration of 40 ug/mL.

The concentration of the active ingredient varied per mL as well as per drop. Therefore, this would lead to varied bioavailability of the active ingredient per drop. This can lead to suboptimal IOP reduction with potential for increase in adverse effects. The concentration per drop for Travatan was 1.01 ug/drop, whereas for TP3 was 1.02 ug/drop and other two drugs ranged between 1.42 and 1.44 ug/drop.

The drop size is also an important consideration. The size of the cul-de-sac is only 7 to 10 μL whereas the drop size of the four travoprost formulations varied from 25.8 to 38.9 μL. The excess medication either may cause a systemic toxicity due to its absorption via the nasolacrimal duct, or may spill over to the eyelid surface leading to increased local side effects, such as hyperemia and skin hyperpigmentation.

Mammo et al^11^ evaluated if brand name glaucoma drops differed from generic equivalents in bottle design, viscosity, surface tension, and volume in North America. For the American brand name Timoptic XE, the average drop volume was 38 ± 3.1 μL *vs* 24 ± 1.5 μL of the generic timolol (p < 0.0001) while that for the Canadian brand name Timoptic XE, the average drop volume was 42 ± 4.0 μL *vs* 25 ± 2 μL of generic timolol (p < 0.0001). For the Canadian brand name Timoptic, the drop volume was 28 ± 1.4 μL *vs* 35 ± 1.9 μL for the generic Timolol (p < 0.01). At a 0.1 per second shear rate, the viscosity of Canadian Timoptic XE was 20 times higher than that of its generic equivalent, whereas the viscosity of American Timoptic XE differed from the generic by a factor of 100. The surface tension of Canadian Timoptic XE was 31% higher than that of the generic (p < 0.001), whereas the surface tension of American Timoptic XE was 21% higher than that of the generic (p < 0.001). The bottle tips of the Canadian and American Timoptic XE were measured about 3.5 times larger than those of their generics. The authors thus concluded that careful consideration should be given to drop viscosity and bottle design when generic ophthalmic products are evaluated for interchangeability and market entry.

In a study done by Johnson et al,^12^ to determine the concentration of bimatoprost, latanoprost, and travo-prost in conditions of simulated daily use and varying degree of thermal stress, medication bottles were stored in calibrated nonhumified, light-free incubators maintained at temperatures below and above the labeled indications for upto 30 days. They concluded that under all combinations of stress “off the shelf” bottles of these prostaglandin analogs showed mean bimatoprost concentration was 102% (100-116%) of the labeled concentration as compared to latanoprost which was 97 to 120% of the labeled concentration and travoprost was 83 to 143% of the labeled concentration. They inferred that higher concentration could be due to evaporation of these drugs in thermal stressed conditions. The results of other studies, namely, Paolera et al,^13^ were in concordance with Johnson et al, where bimatoprost bottles contained concentration of over 100% of the labeled drug concentration when returned 6 weeks after opening, more than 17% of the bottles contained less than 80% of the labeled concentration, and 50% had concentration less than 90% of the labeled drug concentration.

Maintaining the pH of the drug is important as it could affect drug stability and release of active ingredients thereby, affecting the therapeutic efficacy of the drug. Narayanaswamy et al^14^ compared the efficacy and safety of Xalatan with generic latanoprost in patients with primary open-angle glaucoma and found a higher IOP lowering effect of 35% with Xalatan than generic formulation. They also found increased levels of particulate matter and a higher pH compared with the branded drug. There was, however, no significant difference in incidence of conjunctival hyperemia or any other adverse events in both the groups.

This concern of therapeutic equivalence has been a matter of concern, therefore many studies have been reported in literature for various classes of drugs, namely, antibiotics, glaucoma medications, and steroids.^11-18^ In a study, Weir et al,^16^ on comparing the content of cipro-floxacin eye drops, found that three of the five brands of generic ciprofloxacin had significant proportion of sub-optimal concentration. In a number of these preparations, antimicrobial content was low enough to have a potential impact on clinical outcome.

Kahook et al^17^ reported that brand name formulations contained active ingredients and BAK in concentrations that were generally in agreement with their package inserts at baseline. The two generic formulations of latanoprost evaluated were seen to contain baseline levels of active ingredients that were 10% greater than their labeled value. They also found that the generic latanoprost formulations had significant loss of active ingredient concentration after exposure to 25°C and 50°C for 30 days. Benzalkonium chloride concentrations remained stable at 25°C but decreased in some bottles at 50°C. Bottles of both generic medications had higher levels of particulate matter compared to brand name versions.

They therefore concluded that exposure to temperatures at the high end of the labeled value may lead to a significant decrease in concentration of active ingredients in generic formulations that could influence clinical efficacy. They recommended that a reevaluation of IOP lowering efficacy may be indicated in glaucoma patients switching from brand name to generic formulations.

However, no such study has been performed on tra-voprost formulations.

Another study by Velpandian et al,^18^ done at our Centre, was on the stability of latanoprost in generic formulations using controlled degradation and patient usage simulation studies. Extreme pH conditions, oxidation, light, and heat were found to be the significant factors for high degree of latanoprost degradation. We did not evaluate the role of temperature in the present study, although high temperatures during the summer season (45°C) may impact travoprost concentrations.

In conclusion, this pilot study demonstrated significant variations in the physical properties of branded generics. According to the pharmacopeia, the drug concentration should be within 10% of the labeled values, but two of the generic formulations did not meet this criterion. However, we did not perform a clinical correlation of the physical properties and the IOP lowering efficacy and, therefore, cannot comment on the impact of these physical variations in drug properties on the IOP outcomes or the side effects. This study underscores the need for a better quality control, as new generic ocular hypotensive medications become available for public use and, at the same time, reiterates the fact that physical properties of some generic formulations are at par with international standards and may serve as good substitutes for the branded versions. It must be kept in mind that not all generic glaucoma medications are comparable to the branded counterparts, even though they may still be therapeutically effective in many instances.
